# The grading model for the assessment of the total amount of epidural fibrosis in postoperative lumbar spine

**DOI:** 10.1007/s00586-012-2537-z

**Published:** 2012-10-13

**Authors:** Zvonimir Ivan Lubina, Senka Baranovic, Ivan Karlak, Karlo Novacic, Tanja Potocki-Karacic, Dražen Lovrić

**Affiliations:** 1 Department of Diagnostic and Interventional Radiology, University Hospital “Merkur”, Zajčeva 19, 10000 Zagreb, Croatia; 2 Department of Anesthesiology and Intensive Care Unit, University Hospital “Sestre milosrdnice”, Vinogradska 29, 10000 Zagreb, Croatia; 3 Clinic of Traumatology, University Hospital “Sestre milosrdnice”, Vinogradska 29, 10000 Zagreb, Croatia; 4 University Hospital “Sveti Duh”, Sveti Duh 64, 10000 Zagreb, Croatia; 5 Polyclinic “Sunce”, Zagreb, HR 10000 Croatia

**Keywords:** Grading model, Lumbar spine, Failed back surgery, Epidural fibrosis, Magnetic resonance imaging

## Abstract

**Purpose:**

To present a new model derived from Ross’s model for the assessment of the total amount of epidural fibrosis and to present inter- and intravariability study.

**Methods:**

Two readers blinded to each other and blinded to their first and second reading retrospectively evaluated the magnetic resonance examinations in 32 postoperative spine surgery patients using this model.

**Results:**

Paired and unpaired two-sided *t* tests showed no significant difference between the first and second reading, and interclass correlation coefficient revealed good interobserver reliability.

**Conclusion:**

The proposed model enables estimation of the amount of epidural fibrosis in postoperative lumbar spine and does not require any additional software or hardware. It is designed for multi-centered clinical studies where it is necessary to compare the values of epidural fibrosis between the tested and control group. The use of the proposed model is fast and practical and helps to avoid complications arising from image format, calibration and software, which are often encountered in multi-centered studies.

## Introduction

Epidural fibrosis (EF) is a possible postoperative complication after lumbar spine (LS) surgery, in which normal epidural fat is replaced by scar tissue. Patients with EF experience radicular pain 3.2 times more frequently than those without it [1], but there is still some disagreement about whether scar tissue is responsible for recurrent radicular pain or not [2]. A prospective study conducted on 119 patients showed no association between the amount or localization of EF and clinical presentation [3]. EF can be accurately detected with contrast-enhanced magnetic resonance imaging (MRI), which can accurately differentiate it from recurrent disc herniation [4–9]. Contrast-enhanced computed tomography (CT) of the spine or CT myelography is also helpful in the demonstration of EF if MR cannot be performed, but not to the same extent as MRI [10–13]. The epidural space is irregularly filled with ill-defined scar tissue, which can compress or retract the dural sac and/or nerve roots. EF is very commonly found in more than one axial and sagittal slice of MRI.

In the works of Ross et al. [1, 14], the method of estimation of EF in a single slice of MRI is described. This method is, however, based on planar quantification and lacks comprehensive volume information on the extent of the pathological process. The purpose of our study is to investigate the model of fibrosis quantification in more than one adjoined axial MRI slice at a certain level, aimed at predicting the total volume of epidural space affected by fibrosis.

## Materials and methods

**Fig. 1:**
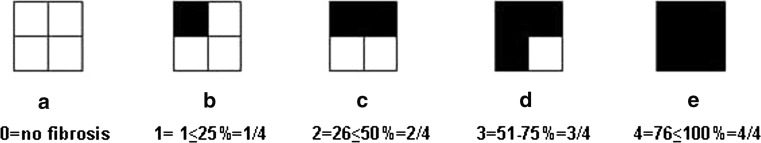
Schematic representation of the range of values and the corresponding surface

The method of EF quantification by MRI in a single slice has been described in detail elsewhere [1, 14]. Briefly, Ross et al. divided the spinal canal into four quadrants by drawing perpendicular lines from the center of the dural sac. Quadrants A and B represent the right and left anterior epidural spaces, respectively, and encompass the lateral recesses and spinal nerve roots. Quadrants C and D represent the right and left posterior epidural spaces, respectively. At the laminectomy level, a posterior border of the evaluation area is defined by drawing the line between the most posterior bony remnants. The authors quantify EF for each of the four quadrants separately, using a scale of 0–4: 0—no/trace EF, 1—1–25 %, 2—26–50 %, 3—51–75 % and 4—76–100 % of quadrant affected by EF (Fig. 1). Therefore, for each operative level, including five imaging slices centered about the intervertebral disc, a minimum of 0 and a maximum of 20 scores can be obtained.

**Fig. 2:**
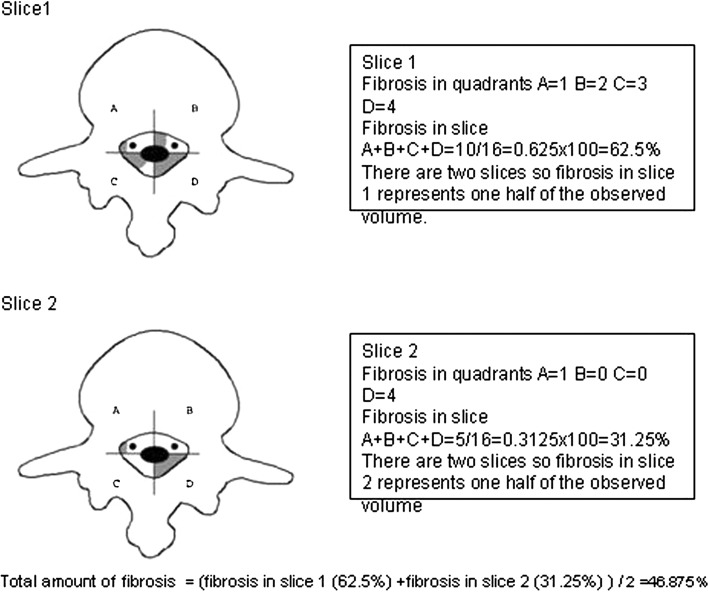
Assessment of fibrosis in the segment using two slices

**Fig. 3:**
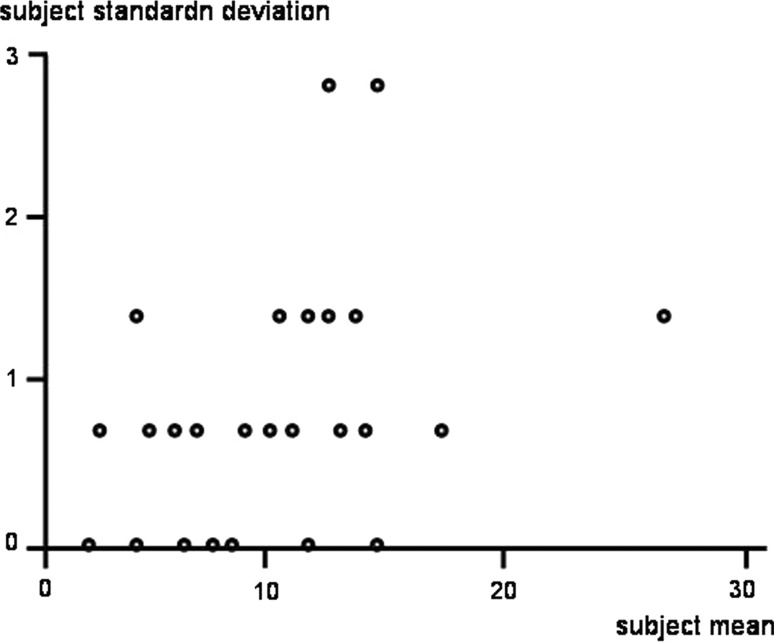
Scatter plot shows repeatability plot

**Fig. 4:**
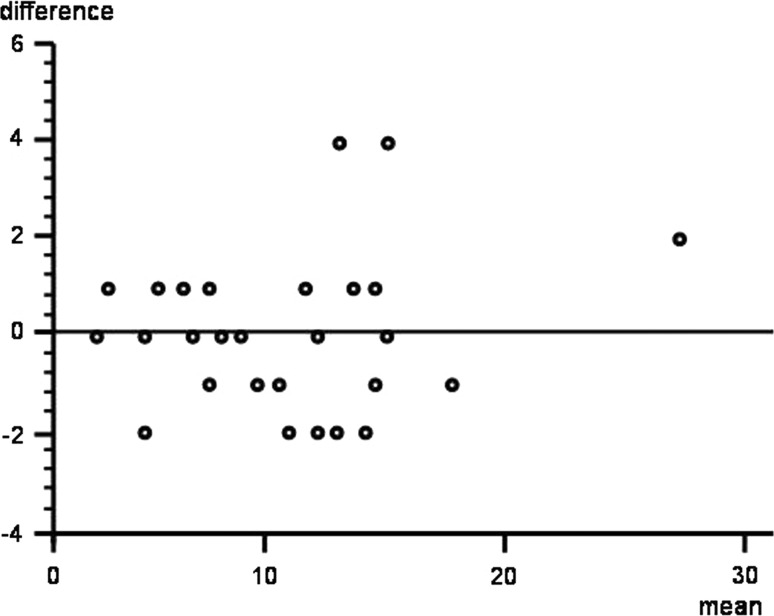
Scatter plot shows agreement plot (95 % limits of agreement)

### Modification of the Ross's method

On analyzing method that Ross et al. [1, 14] used to estimate epidural fibrosis in quadrants, it becomes clear that the exact amount of scar tissue is not relevant. What is important is the range of quadrants affected by epidural fibrosis.

Accordingly, value 1, which represents the amount of fibrosis in the quadrant in the range 1 ≤ 25 %, represents 1/4 of the quadrant affected by fibrosis (Fig. 1b). Value 2, which represents the amount of fibrosis in the quadrant in the range 26 ≤ 50 %, represents 2/4 of the quadrant affected by fibrosis (Fig. 1c). Value 3, which represents the amount of fibrosis in the quadrant in the range 51 ≤ 75 %, represents 3/4 of the quadrant affected by fibrosis (Fig. 1d). Value 4, which represents the amount of fibrosis in the quadrant in the range 76 ≤ 100 %, represents 4/4 of the quadrant affected by fibrosis (Fig. 1e).

If we look at the slice as a whole, then quarters of the quadrants actually represent sixteenths of the slice. As a result, number 1, which in Ross’s model represents 1/4 of the quadrant affected by fibrosis, would in our model correspond to 1/16, or 6.25 % of fibrosis per slice. Accordingly, number 2, which in Ross’s model stands for 2/4 of the quadrant affected by fibrosis, would in our model represent 2/16, or 12.5 % of fibrosis per slice. Number 3 would correspond to 3/16, or 18 % of fibrosis per slice, while number 4 would signify 4/16, or 25 % of fibrosis per slice.

So, the first step in the quantification model that we propose in this paper is to divide the spinal canal into four quadrants in the way proposed earlier in the text. After that, epidural fibrosis is quantified for each quadrant separately as Ross et al. proposed [1, 14].

The values attributed to each quadrant are added, which in turn produces values from 0 to 16 and these values represent the number which shows how much of one-sixteenth of the slice is affected by fibrosis. To get the percentage of the slice affected by fibrosis, the sum thus gained needs to be divided by 16 and multiplied with 100. That way, the percentage of the slice affected by fibrosis is obtained (Table 1).

**Table 1:** Possible approximate values of fibrosis in the entire slice

1 = 6.25 %	2 = 12.5 %
3 = 18.75 %	4 = 25 %
5 = 31.25 %	6 = 37.5 %
7 = 43.75 %	8 = 50 %
9 = 56.25 %	10 = 62.5 %
11 = 68.75 %	12 = 75 %
13 = 81.25 %	14 = 87.5 %
15 = 93.75 %	16 = 100 %

If we wish to estimate the intensity of fibrosis in a segment of spine imaged on MR examination with *n* axial slices, then fibrosis in one slice represents 1/*n* of the observed segment. It is therefore necessary either to divide the value of fibrosis of each slice by number *n* and add it together to get the fibrosis in a segment, or the simpler way is to add together the fibrosis per slice and then divide the sum by the number of slices *n*.

For example, if we observe fibrosis in the segment represented on MRI with two slices, then fibrosis in one slice represents one-half of the observed volume and we have to divide it by 2. If we observe the segment of the spinal canal represented with five slices of MRI, then fibrosis in one slice represents 1/5 of the observed volume. Therefore, fibrosis in one slice gained, as proposed earlier, should be divided by 5 or the values of fibrosis per slice should be added together and divided by 5 to get the total value of fibrosis in the entire segment.

The following example shows the assessment of fibrosis in the segment using two MRI axial slices (Fig. 2).

### MRI study

We retrospectively analyzed all patients who underwent an MRI of the LS spine 6 months or more after the surgery, regardless of the type of pain (radicular or back pain). A total number of 32 patients with EF after laminectomy and discectomy were found. MRI studies were performed on a 1.5 T MR unit (MAGNETOM Symphony, Siemens) using body coil. MR examinations consisted of sagittal and axial T1- and T2-weighted images. After intravenous administration of contrast, sagittal and axial T1-weighted images were repeated within the first 20 min. Gadolinium was administered at a dosage of 0.1 mmol kg^−1^, slow i.v. push. The T1 sagittal sequences were performed using parameters of TR 654 ms/TE 13 ms with fast spin echo (FSE), 35 cm field of view (FOV), a 512 × 224 matrix and 4 mm slice thickness with 0.4 mm spacing. The T2 sagittal sequences were performed using TR 2760 ms/TE 99 ms with FSE, 35 cm FOV, a 512 × 224 matrix, 4 mm slice thickness and 0.4 mm spacing. The T1 axial sequences were performed using TE 353 ms/TR 14 ms with FSE, 26 cm FOV, 320 × 224 matrix, 4 mm slice thickness and 0 mm spacing. The T2 axial sequences were performed using TE 2540 ms/TR 134 ms with FSE, 26 cm FOV, 320 × 224 matrix, 4 mm slice thickness and 0 mm spacing. Five axial slices at the level of laminectomy or discectomy were included in the analysis angulated parallel to the lumbar disc. One slice was performed throughout the disc and two slices above and below the disc. Two radiologists analyzed patients independently on two occasions, with an interval of 3 weeks between the two readings. Scar tissue identification was done on the basis of parameters published elsewhere in studies [1–9, 14, 15]. The proposed modified model was used for estimation of total amount of EF in postoperative LS.

## Results

Each reader analyzed 32 patients with the interval of 3 weeks between two readings (Table 2).

**Table 2:** Reading results

Patient number	1	2	3	4	5	6	7	8	9	10	11	12	13	14	15	16
Radiologist no 1 first reading	11	16	27	2	6	16	6	6	7	2	12	9	14	7	3	8
Radiologist no 1 second reading	13	17	25	2	7	12	6	6	6	2	14	10	14	7	5	8
Radiologist no 2 first reading	14	17	27	2	5	11	9	6	7	5	13	8	13	7	11	6
Radiologist no 2 second reading	13	18	27	2	5	14	10	6	7	4	12	6	13	9	11	6

Patient number	17	18	19	20	21	22	23	24	25	26	27	28	29	30	31	32
Radiologist no 1 first reading	13	5	4	14	6	6	14	9	11	4	10	13	3	11	7	8
Radiologist no 1 second reading	12	4	4	13	6	5	10	11	11	4	12	14	2	10	7	9
Radiologist no 2 first reading	9	5	4	11	6	6	10	10	10	4	13	14	3	11	6	8
Radiologist no 2 second reading	10	5	5	11	8	6	12	8	10	6	12	15	3	11	7	7

Paired, two-sided *t* tests was used to test for the difference between the first and second reading for each radiologist (Table 3). For the first radiologist, *P* value was equal to 0.81 and for the second radiologist, 0.23. The difference was not statistically significant.

**Table 3:** Paired *t* test values

Variability	Mean	SD	SEM	95 % Cl	*P*
Intraobserver					
Radiologist 1	0.06	1.47	0.26	−0.47 to 0.59	0.81
Radiologist 2	−0.25	1.16	0.2	−0.66 to 0.16	0.23

Unpaired two-sided test was used to test the difference between results obtained by the first and the second radiologists in both readings and showed high agreement. *P* value for the first reading was 0.81 and for the second reading, 0.74.

The analysis of reliability between the first and second radiologist revealed good interobserver reliability (ICC 0.95; 95 % CI 0.87–0.97) (Figs. 3, 4).

## Discussion

EF is one of several major causes of failed back surgery. Since the presence of EF makes surgical dissection difficult, lumbar revision surgery bears a high risk of intraoperative complications (e.g., bleeding, nerve root lesions, dural tears). To facilitate tissue dissection and entry into the spinal canal and to reduce the operative complications and surgical time, mucolytic agents for chemical dissection of EF have been developed [16]. In addition, some agents decrease not only the amount of EF, but also the tenacity of EF which makes lumbar revision surgery easier with fewer complications [17]. So far, MRI is the only possible method for evaluation of in vivo efficiency of medicines that inhibit scar tissue growth. To be able to compare the test group and control group, researchers need a model of quantification of the total amount of scar tissue based on MRI.

Multi-center radiological studies commonly include several centers from different parts of the world, so that a wide database of patients could be created. Images are sent to the headquarters from where they are forwarded to the radiologist via a server. This model allows data collection for a large number of patients with specific pathology, which is typically not the case when data collection is restricted to work in one hospital.

Since multi-centered studies rely on databases and images from different clinical centers around the world, images sent to radiologists often come in different formats. For instance, though DICOM is a standardized format used in radiology nowadays, multi-center studies often employ a great number of inadequate and uncalibrated images with no calibration scale or formats, which cannot be uploaded into the existing PACS. Sometimes, images are saved in a format which can only be opened by a particular viewer which does not have a needed calibration option or measurement tool. In such situations, the viewer only allows viewing the image or printing to film.

Due to the above reasons and also due to the nature of EF, which often has unclear boundaries and spreads beyond the spinal canal on the site of laminectomy, the authors wanted to develop a model to help radiologists assess the amount of EF in images which are read without a specialized measuring tool. Namely, the developed model is designed only for clinical studies where it is necessary to compare the values of EF between the tested and control group. The model proposed by the authors is therefore not intended for everyday work activities. This is stressed because our focus in this paper is not on the correlation between EF and clinical status. Similarly, we do not want to highlight the position of EF in the spinal canal, i.e., whether it is anteriorly located (often symptomatic) or posteriorly located (often asymptomatic). Instead, it is our intention to present the developed model and show the correlation between the interobserver and intraobserver reading. The advantage of this «modified» Ross model is that it shows the total amount of spinal canal affected by EF, which allows comparison of the amount of EF between the tested and control group in testing medicines used for EF prevention. The main problem with both models is the precise definition of quadrant boundaries. Since often at the site of laminectomy, EF spreads beyond the spinal canal and EF boundaries are unclear and gradual, the use of measurement software can also be difficult and cumbersome. The additional problem with the modified model we propose in this paper is that if a large number of layers are analyzed and most of them are not affected by fibrosis, the total amount of fibrosis might be underscored. For the same reason, it is necessary for both control and test groups to have the same number of layers, but not more than five as suggested [1, 8].

## Conclusion

This paper proposes modification of Ross et al.’s model of assessment of EF. The proposed model allows estimation of the total amount of EF in the spinal canal after surgery. It is simple for use and does not require any additional software or hardware.
